# A Review of Welding Process for UNS S32750 Super Duplex Stainless Steel

**DOI:** 10.3390/ma17215215

**Published:** 2024-10-26

**Authors:** Tianqing Li, Kai Wang, Yucheng Lei

**Affiliations:** School of Materials Science and Engineering, Jiangsu University, Zhenjiang 212013, China

**Keywords:** super duplex stainless steel, welding, austenite phase, ferrite phase, welded joint

## Abstract

Super duplex stainless steel UNS S32750 is widely used in marine industries, pulp and paper industries, and the offshore oil and gas industry. Welding manufacturing is one of the main manufacturing processes to make material into products in the above fields. It is of great importance to obtain high-quality welded UNS S32750 joints. The austenite content and ferrite content in UNS S32750 play an important role in determining UNS S32750 properties such as mechanical properties and corrosion resistance. However, the phase proportion between the ferrite phase and austenite phase in the welded joint will be changed during welding. Lots of research has been done on how to weld UNS S32750 and how to obtain welded joints with good quality. In this work, the recent studies on welding UNS S32750 are categorized based on the welding process. The welding process for UNS S32750 will be classified as gas tungsten arc welding, submerged arc welding, plasma arc welding, laser beam welding, electron beam welding, friction stir welding, and laser-MIG hybrid welding, and each will be reviewed in turn. The microstructure and properties of the joints welded using different welding processes will also be discussed. The critical challenge of balancing the two phases of austenite and ferrite in UNS S32750 welded joints will be discussed. This review about the welding process for UNS S32750 will provide people in the welding field with some advice on welding UNS S32750 super duplex stainless steel.

## 1. Introduction

Super duplex stainless steel (SDSS) is composed of a ferrite phase and an austenite phase at room temperature. The ferrite phase proportion is approximately 50% while the austenite phase is also about 50% [1,2], which is a very important characteristic of super duplex stainless steel. Due to the specific austenite/ferrite phase ratio, super duplex stainless steel exhibits excellent mechanical properties and corrosion resistance [3,4,5,6]. Super duplex stainless steel can be widely applied in the marine industry, pulp and paper industries, the offshore oil and gas industry, and other places where temperatures are higher or corrosion conditions are more aggressive [7,8,9,10,11]. Welding manufacturing is one of the main manufacturing processes for making super duplex stainless steel materials into products in the above fields [7,12,13,14]. Achieving high-quality welding for super duplex stainless steel is of great importance. One of the key issues in welding super duplex stainless steel is keeping a balance of phase proportions between the ferrite phase and the austenite phase in both the weld zone and heat-affected zone (HAZ). UNS S32750 is a typical representative of super duplex stainless steel. A lot of studies on welding UNS S32750 have been carried out in recent years. It is found that the austenite phase proportion in the weld zone may decrease [10,15,16,17,18], and detrimental intermetallic phases may form [19,20,21,22]. Due to phase proportion changes in welding joints, the performance of UNS S32750 welding joints will be affected [23,24,25,26,27,28,29,30]. In order to know the research and development on welding UNS S32750 systematically and clearly, in this study, the studies on welding UNS S32750 are categorized based on the welding process.

The welding processes for UNS S32750 can be classified as gas tungsten arc welding (GTAW), submerged arc welding (SAW), plasma arc welding (PAW), laser beam welding (LBW), electron beam welding (EBW), friction stir welding (FSW), and laser-MIG hybrid welding. Gas tungsten arc welding, also known as TIG welding, is one of the most commonly used welding techniques for super duplex stainless steels. Recent studies focus on refining the welding parameters to maintain the ideal ferrite/austenite phase balance. This is critical for preserving the corrosion resistance and mechanical properties of SDSS. Nitrogen addition during GTAW has been shown to stabilize the austenite phase and mitigate the loss of corrosion resistance. Submerged arc welding is widely used for thicker sections of SDSS. However, the high heat input of SAW often results in excessive ferrite content, degrading toughness and corrosion resistance. Plasma arc welding is gaining attention due to its precision and control over heat input, making it suitable for thinner sections of SDSS. The heat-affected zones (HAZs) in PAW joints are small, and PAW joints are of good quality. Current research has been investigating the use of PAW for welding SDSS in highly corrosive environments, where phase balance and low HAZs are critical. Laser beam welding can weld thin sections of SDSS with high welding speed. The rapid cooling associated with LBW can result in a high ferrite content, which compromises ductility and corrosion resistance. Electron beam welding offers deep penetration and low heat input, making it ideal for SDSS applications that require minimal thermal distortion. Research in EBW has focused on controlling the ferrite/austenite balance by adjusting the beam power and welding speed. Friction stir welding is a solid-state welding process that has garnered interest for SDSS due to its ability to avoid melting, thereby minimizing phase imbalances and material degradation. Recent research demonstrates that FSW can effectively produce defect-free joints with excellent mechanical properties. However, achieving the correct phase balance remains challenging. Hybrid welding techniques, such as combining laser with arc welding (Laser-GTAW or Laser-MIG), have shown promise in improving the weld quality of SDSS. Research shows that hybrid welding can achieve better control over the austenite/ferrite phase ratio, leading to superior mechanical properties and corrosion resistance. It is of great importance and meaningful to give a review of welding process for UNS S32750 super duplex stainless steel. In this work, the overview and discussion about these welding processes on UNS S32750 will be demonstrated in turn. By this review, it may provide people in the welding field some advice on welding UNS S32750 super duplex stainless steel.

## 2. Recent Researches on Welding UNS S32750

### 2.1. UNS S32750 Welding by GTAW Process

Gas tungsten arc welding (GTAW) refers to a fusion welding method in which the base material is melted by the arc heat generated between the tungsten electrode and the workpiece under the protection of inert gas [31,32,33,34]. Welding speed in GTAW is low [35], but the quality of welded joint by GTAW is good [36,37,38,39,40,41]. GTAW is commonly used to weld UNS S32750.

**Figure 1:**
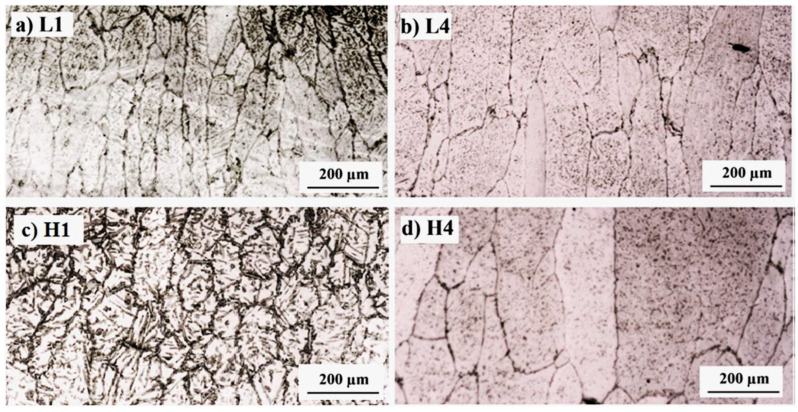
Ferrite grain of weld zone: (**a**) L1, (**b**) L4, (**c**) H1, and (**d**) H4 [16].

The influence of heat input on nitrogen content in the weld of UNS S32750 welding joints created using GTAW is analyzed. Hosseini [16,42,43] conducted a GTAW experiment on UNS S32750 with heat inputs of 0.37 KJ/mm and 0.87 KJ/mm, in order to determine changes in nitrogen content and phase distribution. The results showed that the nitrogen content in welds with a low heat input of 0.37 KJ/mm decreases from 0.28 wt.% to 0.17 wt.% in value, while the nitrogen content in welds with a high heat input of 0.87 KJ/mm decreases more. Table 1 demonstrates the value of nitrogen content, ferrite content, and ferrite grain width in the weld zone. L1, L2, L3, and L4 represent the first, second, third, and fourth weld pass with low heat input, while H1, H2, H3, and H4 represent the first, second, third, and fourth weld pass with high heat input. With high heat input, the weld pool exists for a longer time, and nitrogen has a longer time and more chances to escape. Therefore, the nitrogen content in the weld pool becomes less. With the nitrogen content decreasing, the ferrite grain size becomes larger, as shown in Figure 1. Correspondingly, the ferrite phase proportion in the weld increases more than 20%. As a consequence, the corrosion resistance of the weld is reduced. Nitride is observed near the fusion boundary of the samples with a low heat input of 0.37 KJ/mm, and nitride content does not change with welding pass. No nitride is observed in the first and second weld with a high heat input of 0.87 KJ/mm, and nitride appears after the third pass, and nitride content in the fourth pass increases. The sigma phase generates between 828 °C and 1028 °C.

The effect of N_2_ content on the microstructure of UNS S32750 welded joints by the GTAW process was studied. Du [44] used GTAW on UNS S32750 with different N_2_ contents in shielding gas. The research demonstrated that the amount of austenite in the weld metal increases with the N_2_ content increasing at the range from 0.0% to 5.0%. When the N_2_ content exceeds 2%, the austenite content in the weld is higher than the austenite content in the base plate metal. When the N_2_ content exceeds 3%, the austenite content in HAZ is higher than the austenite content in the base plate metal. The austenite content reaches 51–53% when the N_2_ content ranges from 2.0% to 3.0%. It has also been found that the mechanical properties of the joint are the best when the N_2_ content in the shielding gas reaches the extent of 2.0–3.0%. However, when the N_2_ content exceeds 5.0%, an unstable arc is observed. Thus, according to this study, in order to obtain a stable arc and good quality weld, the N_2_ content in shielding gas should not exceed 5.0%. Westin [45] researched the influence of N_2_ content in back shielding gas on the microstructure of UNS S3275 welded joints.

The active flux tungsten inert gas (A-TIG) welding process is also adopted in welding UNS S32750 [46]. The effects of the flux in, for example, NiO, MoO_3_, and SiO_2_ on the microstructure, mechanical properties, and corrosion resistance of the joints has also been studied.

Heat treatment is used to improve the quality of UNS S32750 joints welded by the GTAW process. It is found that post-weld heat treatment can reduce the ferrite content to 53% and adjust the element distribution [47]. Zhang’s research shows that the HAZ and weld metal have the least ferrite content, and the welded joints have the best pitting corrosion resistance, with heat treatment keeping the temperature at 1080 °C for 3 min [47,48]. Moon [49] studied the influence of heat treatment on σ phase precipitation and pitting corrosion resistance, and found that pitting corrosion generally occurs at the phase boundary between the ferrite and σ phase or the phase boundary between the austenite and σ phase. The influence of the heat treatment temperature on the corrosion mass loss was also reported.

### 2.2. UNS S32750 Welding by SAW Process

**Figure 2:**
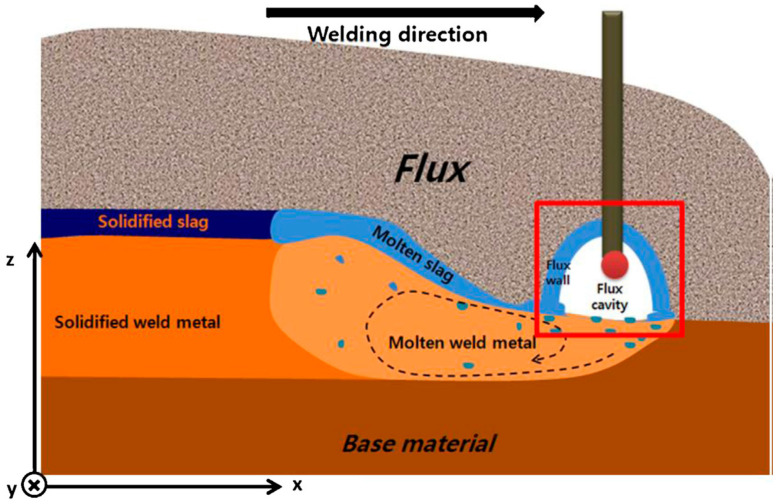
Schematic diagram of SAW [53].

Submerged arc welding (SAW) is a kind of arc welding, using both filler metal and flux. In SAW, the arc burns under the flux layer [50,51,52], as shown in Figure 2 [53]. The heat input in the SAW process is large, and may be good for austenite precipitating from ferrite in the weld, but may induce grain size growing which is not good for the welded joint. Because of the weakness of the SAW process, there are very few applications or studies using the SAW process for welding UNS S32750.

**Figure 3:**
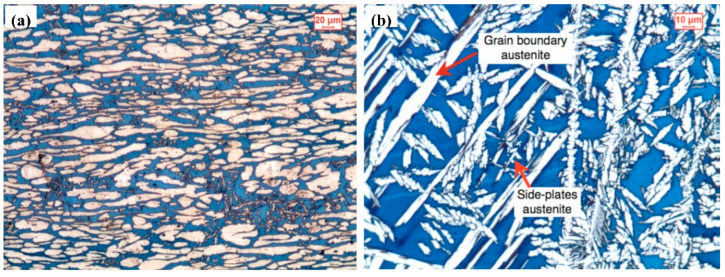
Optical micrographs: (**a**) base metal and (**b**) weld zone [54].

The main issue during the SAW process for UNS S32750 is the high heat input, which can significantly affect the material’s microstructure. This high heat input tends to promote the formation of a higher proportion of ferrite at the expense of the austenite phase, leading to an unbalanced microstructure. To address the issues of phase imbalance and restore corrosion resistance, post-weld heat treatment (PWHT) has been a topic of considerable research. Cervo [54,55] performed post-weld heat treatment on UNS S32750 joints welded by submerged arc welding, and the effect of annealing temperature on the welded joint was studied. The results show that even if the nickel-rich welding wire is added, the austenite content of the welded joint is still lower than that of the base material, as shown in Figure 3. With the post-weld heat treatment kept at 1100 °C for 1 h and then using water quenching, the ratio of the two phases austenite and ferrite is acceptable while the pitting corrosion resistance of the welded joint is good. It can be inferred that the corrosion resistance of SAW welds and mechanical properties of SAW welds in UNS S32750, including tensile strength, impact toughness, and fatigue resistance, are highly dependent on the welding parameters and post-weld treatment.

Research on the welding of UNS S32750 using the SAW process is focused on addressing the key challenges of phase balance, heat input control, and corrosion resistance. By optimizing welding parameters, utilizing alloy-enriched filler materials, and applying appropriate post-weld treatments, it is possible to achieve high-quality welds that retain the excellent properties of super duplex stainless steel. However, it is not a very good choice to conduct SAW in welding UNS S32750. There are few reports referring to UNS S32750 welding by the SAW process.

### 2.3. UNS S32750 Welding by PAW Process

**Figure 4:**
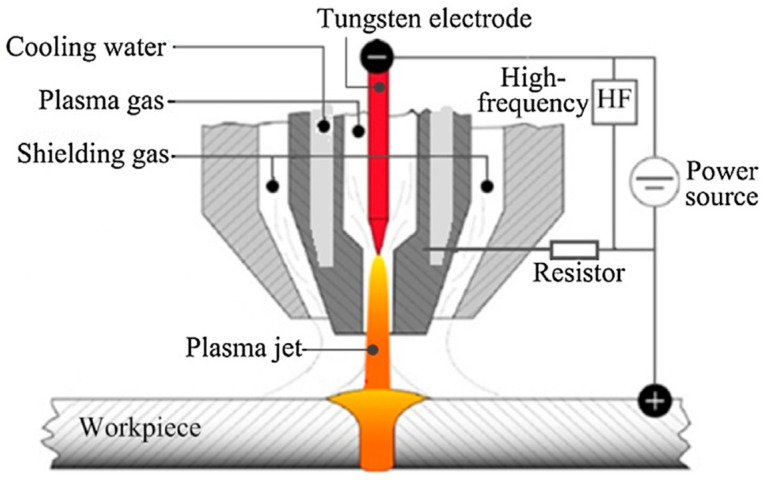
Schematic diagram of PAW [61].

Plasma arc welding (PAW) is an arc welding process with a high energy density [56]. Compared with other free arc welding processes, the arc in PAW is zone constricted, and PAW can fully penetrate workpieces without grooves with one pass while the heat effect on the workpiece is small [57,58,59,60]. Figure 4 is a schematic diagram of PAW [61]. PAW allows for greater control over the heat input, which is critical for maintaining the delicate ferrite-to-austenite phase balance in SDSS. PAW’s concentrated arc results in a more focused heat input, reducing the size of the HAZ. This is important because excessive heat can lead to phase imbalance. Plasma arc welding (PAW) has emerged as a promising technique for welding SDSS, offering enhanced control over the welding process compared to other methods. Therefore, the PAW process is one of the priority options that is used in welding UNS S32750.

The effect of heat input in plasma arc welding on the microstructure and low-temperature toughness of the welded joint was studied. Taban [17] conducted plasma arc welding on UNS S32750 steel plate with different heat inputs, and the relationship between microstructure and mechanical performance was researched. It is found that the welded joints all exhibit good toughness at low temperatures of −20 °C, −40 °C, and −60 °C. Heat input in plasma arc welding has little effect on UNS S32750 steel plate welded joints. However, heat input plays an important role in affecting the ferrite content that in turn affects the mechanical properties of the joint, such as hardness. In Taban’s study, ferrite content decreases with heat input increase, while the hardness of the HAZ increases with heat input increase. The ferrite content in the UNS S32750 steel welded by plasma arc welding reaches 47–54% [62]. It can be inferred that welding parameters, such as welding current, arc voltage, plasma gas flow rate, and welding speed, which affect heat input will influence the ratio of ferrite and austenite remarkably. Maintaining the ideal microstructure in UNS S32750 is critical for ensuring its corrosion resistance and mechanical properties. The PAW process’s precise heat control allows for better management of the ferrite/austenite phase ratio. The use of PAW for UNS S32750 has shown significant potential due to its ability to precisely control heat input, reduce the size of the heat-affected zone, and maintain the delicate phase balance required for SDSS. Ongoing research is focused on optimizing welding parameters, improving microstructural control, and enhancing the mechanical and corrosion properties of PAW welds.

### 2.4. UNS S32750 Welding by LBW Process

Laser beam welding (LBW) is one of the fusion welding processes with a high energy density laser beam as the heat source in welding. Laser beam welding can penetrate fully with lower heat input and higher welding speed, compared to the above arc welding processes such as GTAW, SAW, and PAW [63,64,65,66]. The joint welded by LBW is a good welded joint with small welding deformation and a narrow heat-affected zone [67]. With these advantages, LBW becomes a good choice for welding UNS S32750 steel.

The influence of heat input in laser beam welding on UNS S32750 welded joints has been investigated. Saravanan [68] used a 600 W pulsed Nd: YAG laser power to weld UNS S32750 steel. Three heat input levels, 126 J/mm, 171 J/mm, and 330 J/mm, were used in the research. The hardness and tensile strength of the UNS S32750 welded joint with a heat input of 171 J/mm are higher than with heat input levels of 126 J/mm and 330 J/mm. It was found that the highest hardness occurs on the surface of the joint. In Koleni’s [69] research, the results also show that the heat input plays an important role in influencing the mechanical properties of the UNS S32750 welded joint during laser beam welding.

The effect of post-weld heat treatment on UNS S32750 welded joints by LBW was studied. Saravanan [70] used post-weld heat treatment to keep the temperature at 1050 °C for 2 h, on UNS S32750 welded joints. It was found that the austenite content in the heat-treated joint was increased while the ferrite content in the heat-treated joint was reduced, compared with the as-welded joint. With the post-weld heat treatment, the proportion of the two phases in the joint is close to that in the base metal. Due to the increase in austenite content, the heat-treated joint shows higher corrosion resistance. Post-weld heat treatment is one of the ways to increase the austenite content in UNS S32750 joints welded by laser beam welding and improve the corrosion resistance of UNS S32750 welded joints.

**Figure 5:**
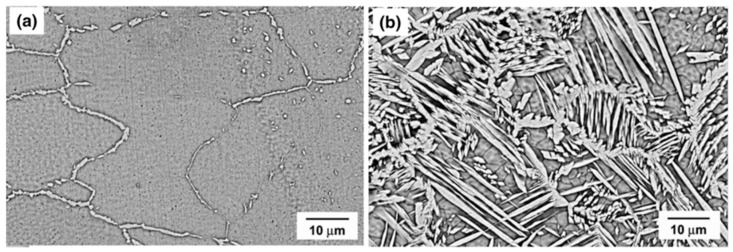
Optical micrographs of weld zone: (**a**) autogenous welding and (**b**) welding with the addition of nickel [18].

In order to increase the austenite content in UNS S32750 welded by LBW, Junior [18] used electrolytic nickel foil as an added metal in laser beam welding for UNS S32750. The results show that the ferrite and austenite contents reach 46.8% and 53.2%, respectively. It is found that the addition of nickel can induce a large amount of intragranular austenite to form, and the two phases, ferrite and austenite, in welds produced by LBW become balanced, as shown in Figure 5. This is a way of increasing the austenite content by adding an austenitizing element such as nickel into the UNS S32750 weld in LBW.

### 2.5. UNS S32750 Welding by EBW Process

**Figure 6:**
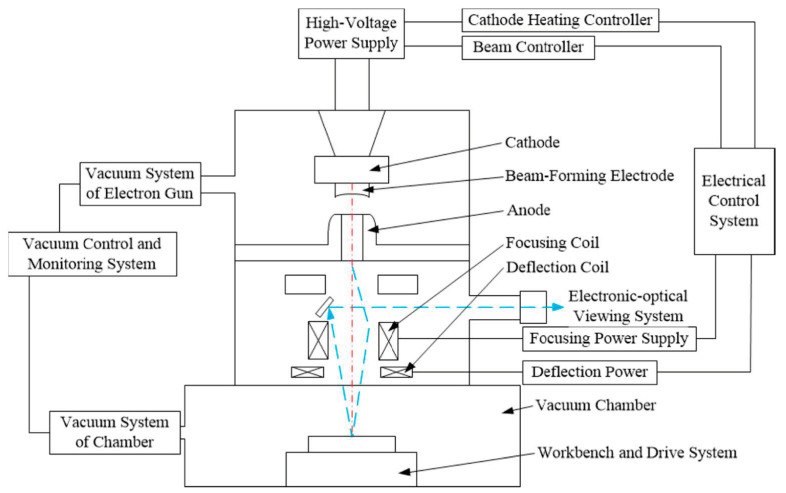
Schematic diagram of EBW [75].

Electron beam welding (EBW) is a high energy density welding process, which can penetrate workpieces with a fast welding speed without grooves [71,72,73]. The electron beam is generated by the electron gun, and is accelerated by the high acceleration voltage, and is focused by the optical system. Then, the electron beam with high speed is used to bombard the workpiece, and finally to melt and vaporize the metal rapidly. The evaporation reaction is the main force for forming the keyhole and achieving the large penetration. The welded joint by EBW is good, and the welding deformation is small while the heat-affected zone is narrow [73,74]. With these advantages, LBW is a good choice for joining UNS S32750 steel. The schematic diagram of EBW is shown in Figure 6 [75].

The weldability of UNS S32750 stainless steel by electron beam welding was studied. Ramkumar [76] conducted EBW on UNS S32750 stainless steel with 6mm thickness and analyzed the weldability of UNS S32750 stainless steel based on metallurgical properties and mechanical properties. The results show that a UNS S32750 stainless steel welded joint of good quality can be obtained by controlling electron beam welding process parameters properly. With the optimized EBW parameters, the ratio of the two phases called austenite and ferrite in the UNS S32750 stainless steel weld is similar to the base metal. The hardness of the weld is higher than that of the base metal UNS S32750 stainless steel, while the tensile strength of the weld is higher than that of the base metal. The electron beam’s power is controlled by adjusting the current and voltage. Researchers are investigating how these variables affect the depth of penetration, phase transformation, and the overall quality of the weld. The focus of the electron beam and the welding speed are critical factors in determining the size of the HAZ and the microstructural changes in the weld. EBW is a promising technique for welding UNS S32750 due to its ability to produce deep, precise welds with minimal heat input. However, maintaining the correct phase balance and preventing the formation of intermetallic phases remain challenges that require the careful optimization of process parameters and post-weld heat treatments.

### 2.6. UNS S32750 Welding by FSW Process

**Figure 7:**
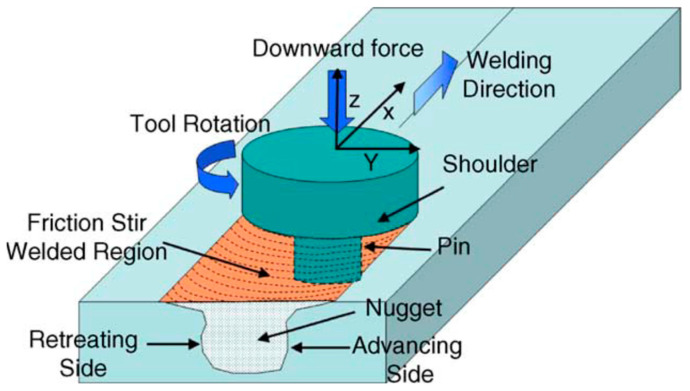
Schematic diagram of FSW [83].

Friction stir welding (FSW) is a kind of solid joining process [77,78]. The shoulder rotates fast or keeps stationary while the pin rotates fast, which makes the metal reach a plastic state [79,80,81,82]. The pin moves forward, and finally the FSW joint is formed. As a solid welding process, FSW is used in welding UNS S32750. The schematic diagram of FSW is shown in Figure 7 [83].

The feasibility of friction stir welding on UNS S32750 has been studied. Giorjão [84] conducted friction stir welding on 8 mm thick UNS S32750 steel pipe. A force control mode of 38 KN was adopted during welding, with a traverse speed of 50 mm/min and rotation speed of 200 rpm. The results show that the hardness of the FSW weld is higher than that of the base metal, reaching about 325 HV. Dynamic recrystallization and grain refinement can be observed at the root of the weld, which is consistent with the results obtained by thermal simulation.

The influence of FSW process parameters on UNS S32750 welded joints has been studied. Mishra [27] applied FSW to welding UNS S32750, and did research on the microstructure evolution and mechanical properties of welded joints. The relationship among FSW process parameters, grain size, and mechanical properties was explored. It was found that the grain size in the stir zone decreases first and then increases with the increase in traverse speed, which is induced by the joint action of heat input, strain rate, and other process parameters. Welded joints with the smaller grain size exhibit better superplastic properties at high temperature. Therefore, for a given rotational speed, other parameters can be controlled to minimize the grain size and improve the mechanical properties of the UNS S32750 welded joint by friction stir welding.

**Figure 8:**
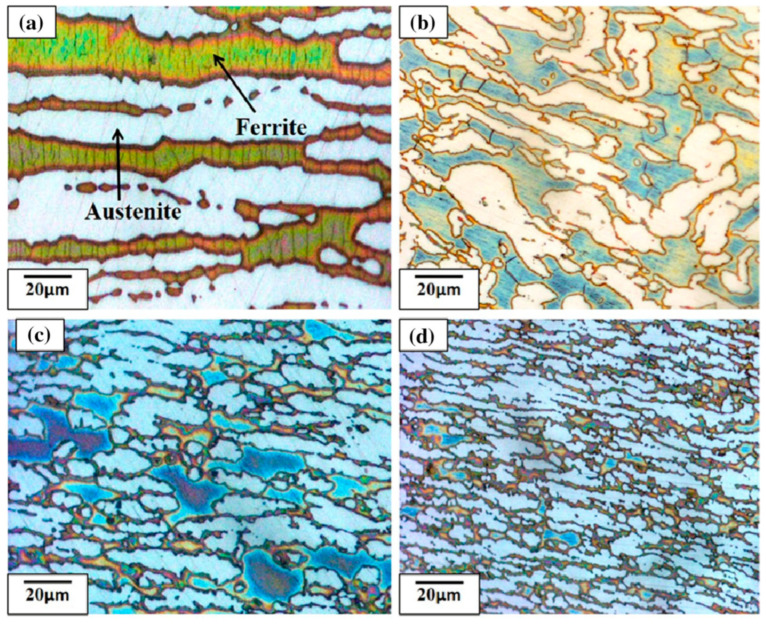
Optical micrographs: (**a**) base metal, (**b**) thermo-mechanically affected zone of one pass, (**c**) stir zone of one pass, and (**d**) stir zone of two passes [85].

The effect of welding passes in FSW on UNS S32750 welded joints is analyzed. FSW can effectively reduce the grain size of ferrite and austenite, and increasing the number of passes will make the grain size smaller, as shown in Figure 8. Increasing the number of friction stir welding passes will improve corrosion resistance [85].

### 2.7. UNS S32750 Welding by Laser-MIG Hybrid Welding Process

**Figure 9:**
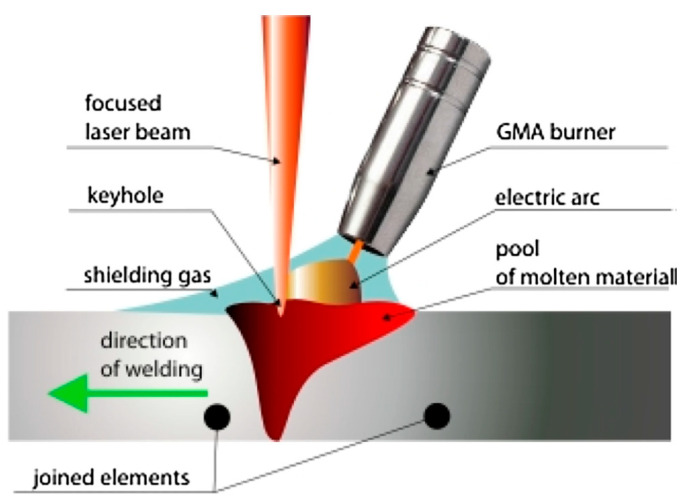
Schematic diagram of laser-MIG hybrid welding [89].

The laser-MIG hybrid welding process is a kind of fusion welding process which combines laser heat and arc heat [86,87,88]. Compared with a one heat source welding method, laser-MIG hybrid welding can make a joint with both laser beam welding advantages and MIG welding advantages. Laser-MIG hybrid welding is a good choice for welding UNS S32750. The schematic diagram of laser-MIG hybrid welding is shown in Figure 9 [89].

Qi [90] conducted a welding experiment on UNS S32750 using a laser-MIG hybrid welding process. The influence of welding parameters on the weld morphology, microstructure, and mechanical properties was studied. It was found that the arc-guided mode laser-MIG hybrid welding with 2 mm distance between the laser and MIG welding electrode can make the coupling action between the laser and the arc appropriate, and reach a good protection effect. The content of austenite in the weld increases with the increase in heat input, and the content of austenite in the weld decreases along the direction of the penetration depth. The tensile strength of the UNS S32750 weld by laser-MIG hybrid welding is higher than that of the base material. It was found that laser power, MIG welding current, welding speed, and shielding gas composition will affect the quality of the UNS S32750 welded joint. Laser-MIG hybrid welding offers significant potential for welding UNS S32750, combining the strengths of both laser and MIG welding to produce high-quality welds with deep penetration, precise heat control, and good phase balance. However, maintaining the delicate ferrite/austenite balance remains a challenge, and ongoing research is focused on optimizing process parameters, filler materials, and post-weld heat treatments to improve the mechanical and corrosion properties of the welds.

A comparison between UNS S32750 welded joints produced by laser beam welding and UNS S32750 welded joints produced by laser-MIG hybrid welding has been made [91]. The ferrite content in LBW joints is higher than that in laser-MIG hybrid welding joints. The pitting corrosion resistance of UNS S32750 welded joints is better in laser-MIG hybrid welding.

To sum up, the main welding processes for welding super duplex stainless steel are GTAW, SAW, PAW, LBW, EBW, FSW, and hybrid welding, as shown in Table 2.

## 3. Critical Challenge in Welding UNS S32750

Keeping the two phases, austenite and ferrite, of welded joints balanced is a critical challenge in welding UNS S32750. The material microstructure of the welded joint will determine the material properties of the welded joint. In the UNS S32750 welded joint, the austenite content and ferrite content play important roles in determining welded joint properties such as mechanical properties and corrosion resistance.

Keeping the two phases of welded joint balanced involves two aspects. One is making the austenite phase and ferrite phase balanced in the UNS S32750 weld, while the other is keeping the austenite phase and ferrite phase balanced in the UNS S32750 heat-affected zone.

In the UNS S32750 fusion weld, the ferrite phase forms firstly during the solidification process, and then the austenite phase is generated from the ferrite phase. In welding, the weld pool cooling rate is high, and therefore the austenite does not have enough time to form. Therefore, the generated austenite phase content in the weld is lower than that in the UNS S32750 base material. There are some solutions to this challenge in the weld. Transmitting austenitizing elements such as nickel and nitrogen to the weld is a preferred choice to improve the austenite content in the UNS S32750 fusion weld. Post-weld heat treatment is also a way to increase the austenite content in the weld.

In the UNS S32750 heat-affected zone, some austenite phase transforms into ferrite phase when the temperature is higher than the ferrite transforming temperature during both the heating process and cooling process, and very little austenite phase is generated from the ferrite phase. The austenite content in the heat-affected zone is decided by the austenite phase decrease because of the austenite phase transforming into ferrite phase and the austenite phase increase because of the austenite phase generated from the ferrite phase. Therefore, it is the solution to the critical challenge in the heat-affected zone, which is how to reduce the austenite phase decrease because of the austenite phase transforming into the ferrite phase and how to increase the austenite phase because of the austenite phase generated from the ferrite phase. Transmitting austenitizing elements are not suitable for improving austenite content in the heat-affected zone. Thus, keeping the austenite phase and ferrite phase balanced in the UNS S32750 heat-affected zone is more difficult than that in UNS S32750 weld. Reducing the width of the heat-affected zone becomes a priority choice. The high energy density welding processes, such as laser beam welding, electron beam welding, and plasma arc welding, with small heat-affected zones, are good for balancing the austenite phase and the ferrite phase in the UNS S32750 heat-affected zone.

Balancing the two phases, austenite and ferrite, of welded joints is one of the most important issues in UNS S32750 welding. Welding metallurgy control and welding parameters adjustment are the main two methods providing solutions to this critical challenge.

## 4. Conclusions

The recent developments regarding UNS S32750 joints welded by different welding processes are reviewed. The UNS S32750 welding processes involve gas tungsten arc welding, submerged arc welding, plasma arc welding, laser beam welding, electron beam welding, friction stir welding, and laser-MIG hybrid welding. It is of great importance to keep the two phases, the austenite phase and the ferrite phase, balanced.

Both welding metallurgy control and welding parameters adjustment are used to balance the austenite phase and the ferrite phase in the UNS S32750 welded joint. It is found that heat input, filler metal, shielding gas, and post-weld heat treatment are the main factors which affect the austenite content, and influence the structure and performance of welded joints. Excessive heat input may cause coarse ferrite grains and precipitate harmful intermetallic phases. If the heat input is too small, the cooling rate will be high. With a high cooling rate, the ferrite does not have enough time to transform into austenite, which makes the ferrite content too high. Adding N_2_ to the shielding gas can increase the austenite content and balance the ferrite/austenite phase ratio, but too much N_2_ may affect the stability of the arc. A reasonable post-weld heat treatment process can adjust the distribution of elements, reduce the ferrite content, and improve the performance of the welded joint. The corrosion resistance of welds and mechanical properties of welds in UNS S32750, including tensile strength, impact toughness, and fatigue resistance, are highly dependent on the phase ratio of ferrite/austenite. Welding methods, welding parameters, and post-weld treatment play an important role in controlling the ferrite/austenite phase ratio.

High energy density welding processes, such as laser beam welding, electron beam welding, and plasma arc welding, are a good way to balance the austenite phase and the ferrite phase in the UNS S32750 heat-affected zone. However, the cooling rate in laser beam welding and electron beam welding is much higher than that in arc welding, which is not good for the austenite phase generated from the ferrite phase. So far, balancing the two phases, austenite and ferrite, of welded joints is still the critical challenge in welding UNS S32750. It is important and meaningful to do further research on welding UNS S32750 super duplex stainless steel.

## Figures and Tables

**Table 1:** Nitrogen content and ferrite content and ferrite grain width of weld zone [16].

	BM	L1	L2	L3	L4	H1	H2	H3	H4
Nitrogen content (wt.%)	0.28	0.23	0.21	0.18	0.17	0.21	0.19	0.13	0.10
Ferrite content (%)	55 ± 3	51 ± 3	57 ± 3	65 ± 3	75 ± 3	51 ± 3	56 ± 3	73 ± 3	79 ± 3
Ferrite grain width (μm)	-	56 ± 5	61 ± 5	92 ± 10	85 ± 3	85 ± 3	105 ± 6	125 ± 10	133 ± 12

**Table 2:** Main welding processes in welding super duplex stainless steel.

Welding Process	Author	Observation
GTAW	Hosseini VA et al. [16,23,42,43]	Compared with base metal, after four passes, the nitrogen content of samples with low and high heat input reduced 0.11 wt.% and 0.17 wt.%, respectively. The nitrogen loss resulted in an increase in nitride precipitates and the content and grain size of ferrite.
	Du DF et al. [44]	The austenite content increases with the increase in the content of N_2_ in shielding gas. When using Ar +2~3% N_2_ as shielding gas, the austenite content was 51~53%.
	Ramkumar KD et al. [30,46]	When employing activated flux tungsten inert gas welding, the addition of flux (NiO, MoO_3_, and SiO_2_) can obtain a complete penetrated joint but has no significant effect on the content and grain size of ferrite.
	Zhang Z et al. [47,48]	Post-weld short duration heat treatment can reduce the ferrite content in the HAZ and WM and adjust the distribution of elements to improve the pitting corrosion resistance of welded joints.
	Moon IJ et al. [49]	A slow cooling rate after post-weld heat treatment may result in the σ phase precipitation in the weld joint, which is harmful to the pitting corrosion resistance.
SAW	Cervo R et al. [54,55]	Even if nickel-rich filler metal is used, the austenite content of weld metal, compared with base metal, is lower due to the high cooling rate of the welding process. Post-weld heat treatment can increase the austenite content and pitting corrosion resistance of the weld joint.
PAW	Taban E et al. [62]	By controlling the heat input, the weld joints can exhibit a reasonable two-phase ratio and good low-temperature toughness. Compared with LBW, the austenite content of PAW joints is higher.
LBW	Saravanan S et al. [68,70]	A fully penetrated and defect-free weld joint can be obtained with high heat input. As the heat input increases, the hardness and tensile strength of the weld joint both increase firstand then decrease. After post-weld short duration heat treatment, the austenite content and pitting corrosion resistance of the weld joint increase while the hardness and tensile strength decrease.
	Da Cruz Junior EJ et al. [18]	When electrolytic nickel foil is used as an added metal, a large amount of intragranular austenite forms in the weld metal and the ferrite–austenite ratio is similar to the base metal.
	Koleni D-IF et al. [69]	By controlling short-term heat input on the weld joint after welding, the ferrite–austenite ratio can be adjusted to a reasonable range.
EBW	Zhang Z.-Q et al. [72], Tóth T. et al. [74]	A sound weld joint can be obtained by EBW. The ferrite–austenite ratio is similar to the base metal. The hardness and tensile strength of the weld joint is higher than that of the base metal while toughness is lower.
FSW	Giorjão RAR et al. [84]	An excellent weld joint without surface defects can be obtained, and grain refinement can be observed at the root of the weld joint due to the low thermal cycle hindering dynamic recrystallization.
	Mishra MK et al. [83,85]	Both ferrite and austenite in the weld joint exhibit obvious grain refinement, and with the increase in the number of welding passes, the grain size of ferrite and austenite may become smaller. The micro-hardness, yield strength, tensile strength, lower ductility, and pitting corrosion resistance of welded joints are better than that of base material.
Hybrid Welding	Qi K et al. [90,91]	Compared with LBW, the laser-MIG hybrid weld metal exhibits a higher austenite content and better pitting corrosion resistance.
